# The Anti-Angiogenic Effects of Anti-Human Immunodeficiency Virus Drugs

**DOI:** 10.3389/fonc.2020.00806

**Published:** 2020-05-21

**Authors:** Giovanni Barillari

**Affiliations:** Department of Clinical Sciences and Translational Medicine, University of Rome Tor Vergata, Rome, Italy

**Keywords:** tumor vasculature, angiogenesis, vasculogenesis, HIV-protease inhibitors, HIV-reverse transcriptase inhibitors, CXCR4 antagonists, AKT

## Abstract

The growth and metastasis of malignant tumors benefit from the formation of blood vessels within the tumor area. There, new vessels originate from angiogenesis (the sprouting of pre-existing neighboring vessels) and/or vasculogenesis (the mobilization of bone marrow-derived endothelial cell precursors which incorporate in tumor vasculature and then differentiate into mature endothelial cells). These events are induced by soluble molecules (the angiogenic factors) and modulated by endothelial cell interactions with the perivascular matrix. Given angiogenesis/vasculogenesis relevance to tumor progression, anti-angiogenic drugs are often employed to buttress surgery, chemotherapy or radiation therapy in the treatment of a wide variety of cancers. Most of the anti-angiogenic drugs have been developed to functionally impair the angiogenic vascular endothelial growth factor: however, this leaves other angiogenic factors unaffected, hence leading to drug resistance and escape. Other anti-angiogenic strategies have exploited classical inhibitors of enzymes remodeling the perivascular matrix. Disappointingly, these inhibitors have been found toxic and/or ineffective in clinical trials, even though they block angiogenesis in pre-clinical models. These findings are stimulating the identification of other anti-angiogenic compounds. In this regard, it is noteworthy that drugs utilized for a long time to counteract human immune deficiency virus (HIV) can directly and effectively hamper molecular pathways leading to blood vessel formation. In this review the mechanisms leading to angiogenesis and vasculogenesis, and their susceptibility to anti-HIV drugs will be discussed.

## Introduction

Human Immunodeficiency Virus (HIV) is the etiologic agent of Acquired Immune Deficiency Syndrome (AIDS), a deadly disease characterized by a profound upheaval of the immune system and the consequent increased risk of developing infectious illnesses and tumors (1).

HIV life cycle is mediated by cellular and viral proteins: among the latter, the reverse transcriptase, integrase and aspartyl protease enzymes are key for HIV replication and infectivity (2).

Briefly, HIV infection begins when the gp120 protein of the viral envelope binds to the CD4 receptor on T cell surface (2). This is followed by gp120 interaction with a co-receptor (most often a chemokine receptor), and HIV entry into the cell (2). Thereafter, the HIV reverse transcriptase copies the viral RNA genome into the viral DNA, which is integrated into host cell genome by the HIV integrase, and then transcribed into messenger RNA (2). Subsequently, HIV transcripts are translated into HIV envelope proteins and the fused precursors of HIV capsid and polymerase proteins: this gives rise to the production of immature, non-infectious viral particles that “bud” from infected cells (2). Finally, the HIV aspartyl protease cleaves the fused capsid-polymerase proteins into the functional polypeptides, and HIV matures becoming infectious (2).

Survival of HIV-positive individuals has been greatly extended by the combined Anti-Retroviral Therapy (cART), which has rendered HIV infection a chronic disease (3).

cART results from the mix of drugs inhibiting the HIV reverse transcriptase, integrase or aspartyl protease (3).

In particular, HIV-reverse transcriptase inhibitors halt the synthesis of viral DNA (3), and are available in three forms: (a) nucleoside analog reverse transcriptase inhibitors, which are modified deoxynucleotides analogs competing with natural deoxynucleotides for incorporation in the HIV DNA that is being synthesized; (b) nucleotide analog reverse transcriptase inhibitors, that are phosphonate deoxynucleotides analogs again competing the incorporation of natural deoxynucleotides in the forming HIV DNA; (c) non-nucleoside reverse transcriptase inhibitors, which bind to the allosteric sites of HIV-reverse transcriptase, thereby hampering its function (Table 1) (3). Nowadays, a HIV-reverse transcriptase inhibitors-based cART containing the nucleotide analog tenofovir, the nucleoside analog emtricitabine and the non-nucleoside efavirenz is recommended as first-line treatment of HIV infection (6).

**Table 1:** Anti-HIV drugs approved for clinical use, and their mechanism of action.

**Class**	**Names**	**Mechanism of action**
Nucleoside analog of HIV-reverse transcriptase inibitors	Zidovudine, stavudine, lamivudine, abacavir, emtricitabine	Compete with natural deoxynucleotides for incorporation in the forming HIV DNA (3)
Nucleotide analog of HIV-reverse transcriptase inibitors	Tenofovir, adefovir	Compete with natural deoxynucleotides for incorporation in the forming HIV DNA (3)
Non-nucleoside HIV-reverse transcriptase inhibitors	Efavirenz, nevirapine, delavirdine, etravirine, rilpivirine, doravirine	Hamper the function of HIV-reverse transcriptase by binding to its allosteric sites (3)
Integrase strand transfer inhibitors	Raltegravir, eviltegravir, dolutegravir	Bind to the active site of HIV integrase, thereby inhibiting its function (3)
HIV-protease inhibitors	Saquinavir, indinavir, ritonavir, nelfinavir, amprenavir, atazanavir, darunavir	Mimic the substrate of HIV protease (4)
CXCR4 antagonists	AMD3100	Compete either HIV or CXCL12 binding to CXCR4 (5)

*Here are listed the names, class and mechanism of action of anti-HIV drugs approved for clinical use. In square brackets are references with specific information*.

Among the antagonists of HIV integrase, the integrase strand transfer inhibitors impair HIV replication by preventing the insertion of HIV DNA into the host cell genome (Table 1): these drugs are in established use, especially in patients who have acquired resistance to other cART components (3).

For their part, HIV-protease inhibitors block the active site of HIV aspartyl protease, thus impeding the processing of HIV capsid-polymerase polyproteins and, consequently, the generation of mature, infectious HIV particles (4) (Table 1). To date, 10 HIV-protease inhibitors have been approved for therapeutic use in humans: saquinavir, indinavir, ritonavir, nelfinavir, lopinavir, amprenavir and its derivate fosamprenavir, atazanavir, tipranavir, and darunavir: all of them (but tipranavir) mimic HIV protease substrate (4). At the present time, darunavir or atazanavir are the most used HIV-protease inhibitors: whereas, saquinavir and indinavir are no longer employed because of their low solubility, and ritonavir is used only to increase the half-life of other HIV-protease inhibitors, due to its capability of inhibiting cytochrome P450 3A4 (4).

Consistent with chemokine receptor capability of working as HIV co-receptors, the CXC chemokine receptor 4 (CXCR4) antagonist AMD3100 efficiently reduces virus entry into target cells (Table 1), and it is then administered to HIV-positive individuals (5).

As such, these therapeutic regimens efficiently suppress HIV replication, thus improving the patients' immune functions (3).

Strikingly, results from epidemiological studies indicate that treatment of HIV-infected individuals with HIV-protease inhibitors and/or HIV-reverse transcriptase inhibitors has also reduced the incidence and/or clinical progression of AIDS-defining tumors such as Kaposi's sarcoma, non-Hodgkin lymphoma or uterine cervical carcinoma (7, 8). Moreover, chemokine receptor antagonists have been reported to be effective in the treatment of non-AIDS defining hematological malignancies affecting HIV-infected patients (9).

Definitely, the anti-tumor effects of cART are helped by the capability that this therapeutic regimen has to promote immune reconstitution by suppressing HIV replication (7, 8). Nevertheless, drugs present in cART and employed in the treatment of HIV-positive, immune-deficient patients have been found to exert anti-tumor activities also in HIV-negative, immune-competent individuals (7, 8, 10–22). Moreover, the HIV-protease inhibitors, HIV-reverse transcriptase inhibitors or chemokine receptor antagonists have been shown to directly impair the survival, growth, invasion and/or locomotion of tumor cells in HIV-free pre-clinical models (23–39). Interestingly, in some cancer cell types the cytostatic effect of HIV-protease inhibitors or HIV-reverse transcriptase inhibitors has been accompanied by the induction of cell differentiation and, eventually, by the acquisition of an immunogenic phenotype (7, 30, 31, 40). In addition, the inhibitors of HIV protease or reverse transcriptase and the antagonists of chemokine receptors have been reported to sensitize cancer cells to anti-tumor chemotherapeutics or ionizing radiations both *in vitro* and *in vivo* (32, 41–48).

In contrast, it is not currently known whether the inhibitors of HIV-integrase possess direct anti-tumor activities. Furthermore, the impact that these drugs may have on cancer incidence or progression has not been clearly established (49, 50).

The mechanisms responsible for the anti-tumor activities of HIV protease inhibitors, HIV reverse transcriptase inhibitors or chemokine receptor antagonists include the block of signaling pathways, transcription factors, enzymes, cytokines or growth factors which are deeply involved in tumor development and/or progression (23–39, 41–48).

Noteworthy, many the abovementioned molecules or mechanisms are employed by endothelial or stromal cells to generate blood vessels (51). Consistently, the inhibitors of HIV protease or reverse transcriptase and the antagonists of chemokine receptors have also been shown to counteract tumor vascularization in a variety of pre-clinical models.

In particular, results from early animal studies have indicated that the HIV protease inhibitors indinavir or saquinavir can directly block angiogenesis, that is the sprouting of new blood vessels from pre-existing ones (52). Later, also other HIV protease inhibitors including ritonavir, nelfinavir or amprenavir have been found capable of inhibiting angiogenesis *in vivo* (53, 54), and the anti-angiogenic effect of indinavir or saquinavir has been confirmed in mouse xenografts of highly prevalent human tumors (27). In the meantime, *in vitro* work has unraveled some of the molecular mechanisms responsible for the anti-angiogenic effect of HIV-protease inhibitors (55–59).

Studies evaluating the impact of HIV-reverse transcriptase inhibitors on angiogenesis are more recent than those concerning HIV-protease inhibitors. Results from those studies indicate that HIV-reverse transcriptase inhibitors including zidovudine, stavudine, efavirenz, lamivudine, emtricitabine, abacavir or tenofovir hamper endothelial cell survival, growth and locomotion *in vitro* and angiogenesis *in vivo* (60–63).

At variance with the inhibitors of the HIV protease or reverse transcriptase, the effect that HIV integrase inhibitors could have on angiogenesis has not yet been evaluated.

The discovery of the anti-angiogenic activity of chemokine receptor antagonists is quite novel. In particular, studies on this topic mostly refer to CXCR4 that, in addition to work as a co-receptor for HIV entry into target cells (5), is bound by the pro-angiogenic CXC chemokine ligand 12 (CXCL12) (64, 65). Consistent with the fact that both CXCR4 and CXCL12 are highly expressed in tumor tissues where their interaction plays a major role in the formation of new vessels, the CXCR4 antagonist AMD3100, which is employed in anti-HIV therapies, can counteract angiogenesis either *in vitro* or in animal models of human tumors (64–67).

Given that newly formed blood vessels nourish the growing cancer mass and furnish a portal for its metastasis, the anti-angiogenic properties of anti-HIV drugs constituting cART are likely to strongly contribute to the anti-tumor activity of this curative procedure (7, 8, 62, 68).

This considered, the present review is focused on the cellular events or molecular pathways which make HIV-protease inhibitors, HIV-reverse transcriptase inhibitors or CXCR4 antagonists capable of impairing the formation of new vessels that accompanies and favors tumor progression.

## Effect of HIV-Protease Inhibitors or HIV-Reverse Transcriptase Inhibitors on Pro-Angiogenic Signaling Pathways

In the adult organism, endothelial cells lining the blood vessel lumen have a low proliferative rate: this is due to their tight intercellular junctions or anchorage to the basement membrane, and to the cytostatic stimulus they receive from vascular smooth muscle cells or pericytes surrounding the vessel externally (69, 70). Under these condition, endothelial cells can remain quiescent for years.

However, upon tissue hypoxia or damage and inflammation, endothelial cells are activated and new vessels are eventually formed. In most cases, this occurs through angiogenesis, a multi-step process affecting pre-existing neighbor vessels: there, vascular smooth muscle cells are detached and endothelial cells degrade the basement membrane, migrate in the perivascular space and proliferate, forming cellular cords that will ultimately differentiate into hollow tubes (69, 70).

Hypoxia and inflammation can occur at the same time in tumors. In particular, as the tumor grows, cells located in its central regions become distant to the neighboring vessels, being deprived of oxygen or nutrients, and undergo necrosis (69).

**Figure 1:**
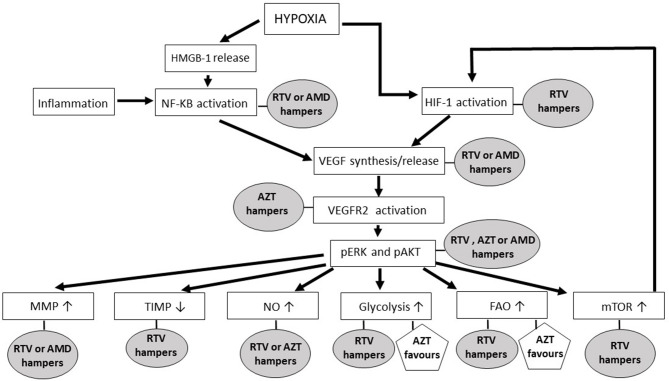
The impact of anti-HIV drugs on the molecular pathways of angiogenesis. In the rectangles are shown the main signaling pathways and molecules leading to new blood vessel formation; in ellipses and pentagons are reported the antagonist and agonist effects, respectively, that anti-HIV drugs exert on the angiogenic molecules or pathways. Ritonavir has been selected among the HIV-protease inhibitors, while zidovudine is the representative of the HIV-reverse transcriptase inhibitors, because both of these drugs are endowed with several anti-angiogenic activities. The chemokine receptor antagonist AMD3100 has been mentioned since it is currently employed in either anti-HIV or anti-cancer therapy. AMD, AMD3100; AZT, zidovudine; FAO, fatty acids oxidation; HIF, hypoxia-inducible factor; HMGB, high mobility group box; MMP, matrix metalloproteinase; mTOR, mammalian-target-of-rapamycin; NF-kB, nuclear factor-kappa B; NO, nitric oxide; pAKT, phosphorylated protein kinase B; pERK, phosphorylated extracellular-signal-regulated kinases; RTV, ritonavir; TIMP, tissue inhibitor of matrix metalloproteinase; VEGF, vascular endothelial growth factor; VEGFR2, type 2 vascular endothelial growth factor receptor.

Necrotic tumor cells release the high-motility-group-box 1 nuclear protein, which induces the Nuclear Factor-kappa B (NF-kB) transcription factor to activate the expression of inflammatory chemokines (71) (Figure 1). The latter, in turn, recruit to the tumor site macrophages and leukocytes releasing pro-angiogenic factors (69). In this regard, it has to be highlighted that HIV-protease inhibitors including lopinavir, nelfinavir or ritonavir can impair NF-kB activation in endothelial cells (72) (Figure 1, Table 2). Moreover, saquinavir, indinavir and atazanavir are capable of inhibiting both the expression of high-mobility-group-box 1 protein and the activation of NF-kB (73, 76, 98) (Table 2). In contrast, the HIV-reverse transcriptase inhibitors tenofovir and zidovudine have no effect on NF-kB activity (101), while efavirenz even increases it (102) (Table 3).

**Table 2:** Anti-angiogenic effect of HIV-protease inhibitors: molecular targets.

**HIV protease inhibitor**	**Mechanism(s) of action and molecular targets**	**Experimental models**
Indinavir	**Effect on angiogenic molecules**: NF-kB, HMGB-1, MAPK or MMP inhibition. eNOS shutdown in some cell types but not in others. No effect on VEGF, AKT, mTOR or GLUT-1.	Pre-clinical (*in vitro* and *in vivo*) and clinical (27, 52, 57, 73–75)
	**Effect on anti-angiogenic molecules**: TIMP up-regulation	
Saquinavir	**Effect on angiogenic molecules**: NF-kB, HIF-1, MAPK, AKT, eNOS or MMP inhibition. Increase in mTOR.	Pre-clinical (*in vitro* and *in vivo*) and clinical (11, 27–29, 52, 72, 76–80)
	**Effect on anti-angiogenic molecules**: no effect on TIMPs	
Ritonavir	**Effect on angiogenic molecules**: NF-kB, HIF-1, VEGF, MAPK, AKT, eNOS, mTOR, MMP, GLUT-1 or FAO inhibition.	Pre-clinical (*in vitro* and *in vivo*) and clinical (23, 28, 29, 53, 59, 72, 77, 79–88)
	**Effect on anti-angiogenic molecules**: TIMP up-regulation	
Nelfinavir	**Effect on angiogenic molecules**: NF-kB, HIF-1, VEGF, AKT, eNOS, or mTOR inhibition. MMP or MAPK inhibition in some cell types, but not in others. No effect on FAO.	Pre-clinical (*in vitro* and *in vivo*) and clinical (23, 54, 55, 72, 75, 77, 80, 86, 89–95)
	**Effect on anti-angiogenic molecules**: TIMP up-regulation	
Lopinavir	**Effect on angiogenic molecules**: NF-kB, MAPK, AKT, eNOS, MMP, or FAO inhibition. No effect on mTOR.	Pre-clinical (*in vitro* and *in vivo*) (12, 25, 58, 83, 96, 97)
	**Effect on anti-angiogenic molecules**: no effect on TIMPs	
Atazanavir	**Effect on angiogenic molecules**: NF-kB, HMGB-1, eNOS or FAO inhibition. No effect on MAPK or GLUT-1.	Pre-clinical (*in vitro*) (58, 88, 98)
	**Effect on anti-angiogenic molecules**: unknown	
Amprenavir	**Effect on angiogenic molecules**: HIF-1, VEGF, AKT, MMP, or eNOS inhibition. No effect on NF-kB.	Pre-clinical (*in vitro* and *in vivo*) and clinical (54, 79, 99)
	**Effect on anti-angiogenic molecules**: unknown	
Darunavir	**Effect on angiogenic molecules**: MMP or FAO inhibition. No effect on NF-kB, AKT, or eNOS.	Pre-clinical (*in vitro*) (88, 100)
	**Effect on anti-angiogenic molecules**: unknown	

*Here are summarized the effects that HIV-protease inhibitors approved for clinical use have on molecules triggering or arresting angiogenesis. AKT, protein kinase B; eNOS, endothelial nitric oxide synthase; FAO, fatty acids oxidation; GLUT, glucose transporter; HIF, hypoxia inducible transcription factor; HMGB, high-motility-group-box; MAPK, mitogen-activated protein kinase; MMP, matrix metalloproteinase; mTOR, mammalian-target-of-rapamycin; NF-kB, Nuclear Factor-kappa B; TIMP, tissue inhibitor of matrix metalloproteinase; VEGF, vascular endothelial growth factor. In square brackets are references with specific information*.

**Table 3:** Effects of HIV-reverse transcriptase inhibitors or AMD3100 on angiogenesis regulators.

**Drug**	**Class**	**Mechanism(s) of action and molecular targets**	**Experimental models**
Zidovudine	Nucleoside analog RTI	**Effect on angiogenic molecules**: VEGFR2, FGFR, MAPK, AKT or eNOS shutdown. MMP down-regulation in some cell types but not in ECs. No effect on NF-kB. Stimulation of glycolysis and FAO.	Pre-clinical (*in vitro* and *in vivo*) and clinical (62, 101, 103–107)
		**Effect on anti-angiogenic events**: increase in EC apoptosis	
Lamivudine	Nucleoside analog RTI	**Effect on angiogenic molecules**: VEGFR2, FGFR, MAPK, AKT, or eNOS shutdown. MMP up-regulation.	Pre-clinical (*in vitro* and *in vivo*) (60, 62, 103, 108)
		**Effect on anti-angiogenic events**: increase in EC apoptosis, TIMP down-regulation	
Stavudine	Nucleoside analog RTI	**Effect on angiogenic molecules**: increase in FAO. No effect on AKT.	Pre-clinical (*in vitro*) (60, 103, 107)
		**Effect on anti-angiogenic events**: unknown	
Emtricitabine	Nucleoside analog RTI	**Effect on angiogenic molecules**: eNOS stimulation.	Pre-clinical (*in vitro* and *in vivo*) (6, 63)
Efavirenz	Non-nucleoside RTI	**Effect on angiogenic molecules**: NF-kB, MAPK, or eNOS activation. Increase in glycolysis and vessel permeability. No effect on AKT.	Pre-clinical (*in vitro* and *in vivo*) (6, 61, 102, 109, 110)
		**Effect on anti-angiogenic events**: increase in EC apoptosis	
Nevirapine	Non-nucleoside	**Effect on angiogenic molecules**: no effect on AKT.	Pre-clinical (*in vitro*) (83)
Tenofovir	Nucleotide analog RTI	**Effect on angiogenic molecules**: VEGFR2, FGFR, MAPK, or AKT shutdown. No effect on NF-kB. eNOS stimulation.	Pre-clinical (*in vitro* and *in vivo*) (6, 62, 63, 101)
AMD3100	CXCR4 antagonist	**Effect on angiogenic molecules**: NF-kB, MAPK, AKT, MMP, or VEGF down-regulation.	Pre-clinical (*in vitro* and *in vivo*) (64, 67, 68, 111)

*Here are summarized the effects that anti-HIV compounds approved for clinical use, including the HIV-reverse transcriptase inhibitors or the CXCR4 antagonist AMD3100, have on molecules triggering or arresting angiogenesis. AKT, protein kinases B; EC, endothelial cell; eNOS, endothelial nitric oxide synthase; FAO, fatty acids oxidation; FGFR, fibroblast growth factor receptor; MAPK, mitogen-activated protein kinase; MMP, matrix metalloproteinase; NF-kB, Nuclear Factor-kappa B; RTI, reverse transcriptase inhibitors; TIMP, tissue inhibitor of matrix metalloproteinase; VEGF, vascular endothelial growth factor; VEGFR2, type 2 vascular endothelial growth factor receptor. In square brackets are references with specific information*.

In tumor central areas also the hypoxia inducible transcription factor (HIF)-1 is activated, directly promoting the expression of angiogenic factors (69, 112) (Figure 1). While HIV-reverse transcriptase inhibitor impact on HIF-1 is currently uncharted, the HIV-protease inhibitors amprenavir, nelfinavir, saquinavir or ritonavir are known to down-regulate HIF-1 expression in both normal and tumor cells (54, 55, 59) (Figure 1, Table 2).

Vascular endothelial growth factor (VEGF)-A, basic fibroblast growth factor, platelet derived growth factor-BB and angiopoietin−1 and−2 are among the angiogenic factors produced by cancer cells, stromal cells or tumor-infiltrating immune cells upon HIF or NF-kB activation (69). By promoting vasodilatation and vascular smooth muscle cell detachment, angiopoietin-2 triggers the initiation of angiogenesis; whereas, angiopoietin-1 or platelet derived growth factor-BB mediates its termination, and VEGF-A or basic fibroblast growth factor promotes all the steps of the angiogenic process (69). Actually, the principal mediator of tumor angiogenesis is VEGF-A, which is produced either in inflammatory-driven or ischemia-induced angiogenesis, since it has response elements for both NF-kB and HIF-1 in its promoter region (69) (Figure 1). Consistent with their inhibitory effect on HIF-1 and NF-kB, the HIV-protease inhibitors ritonavir, nelfinavir or amprenavir reduces VEGF production (54, 55, 59, 77), while indinavir cannot (113) (Figure 1, Table 2).

Angiogenic factors exert their actions by binding to specific receptors whose intracellular domain contains a tyrosine kinase that auto-phosphorylates upon ligand binding (114). The most important receptor in angiogenesis is the type 2 VEGF receptor (114): noteworthy, HIV-reverse transcriptase inhibitors including zidovudine, tenofovir and lamivudine block the activation of either type 2 VEGF receptor or basic fibroblast growth factor receptor in endothelial cells (62) (Figure 1, Table 3).

The binding of an angiogenic factor to its receptor activates signaling pathways such as the mitogen-activated protein kinases (MAPK)/extracellular-signal-regulated kinases (ERK) and/or the phosphoinositide 3 kinase (PI3K)/ protein kinase B (AKT) (114) (Figure 1).

Activation of the MAPK/ERK pathway leads to endothelial cell proliferation (115). The HIV-reverse transcriptase inhibitors zidovudine, lamivudine or tenofovir weaken ERK phosphorylation promoted in endothelial cells by VEGF-A (62) (Figure 1); whereas, efavirenz activates MAPK in endothelial cells (102) (Table 3).

The HIV-protease inhibitors ritonavir, saquinavir, lopinavir or indinavir have definitely proven to counteract this signaling pathway (74, 77, 96), while nelfinavir inhibits it in some cell types but not in others (89, 90) (Figure 1, Table 2).

The AKT cascade is activated by PI3K, an enzyme having different isoforms of whom p110α is selectively required for angiogenesis (112).

PI3K generates the phosphatidylinositol (3,4,5)triphosphate which recruits AKT to the cellular membrane: there, the phosphoinositide dependent kinase-1 activates AKT by phosphorylating its kinase domain (112).

While the HIV-reverse transcriptase inhibitors zidovudine, tenofovir and lamivudine depress VEGF-promoted AKT phosphorylation in endothelial cells (62) (Figure 1, Table 3), the HIV-protease inhibitors nelfinavir, lopinavir and ritonavir decrease both total and phosphorylated AKT levels (54, 55, 81–83, 90–93, 116) (Figure 1, Table 2). Noteworthy, nelfinavir, saquinavir and amprenavir inhibit AKT phosphorylation also in leukocytes of treated individuals (78). In contrast, the HIV-protease inhibitor indinavir and the HIV-reverse transcriptase inhibitors nevirapine or efavirenz have no significant effect on AKT (83, 109, 117) (Tables 2, 3). Notwithstanding, and also in spite of its capability of promoting the activation of either NF-kB or MAPK (102), efavirenz possesses anti-angiogenic activities: in particular, this HIV-reverse transcriptase inhibitor has been found to counteract endothelial cell viability and growth via an increase in oxidative stress (61).

Results from *in vitro* experiments indicate that the HIV protease inhibitor nelfinavir can impair AKT phosphorylation without increasing the expression or activity of AKT negative regulators such as phosphatase-and-tensin-homolog or the protein-phosphatase-2A (94, 95). Interestingly, when combined with the anti-diabetes drug metformin, nelfinavir reduces PI3Kp110α protein levels in tumor cells (118).

AKT phosphorylation promotes endothelial cell expression of endothelial nitric oxide synthase (eNOS), this leading to the production and release of nitric oxide (112) (Figure 1). The latter is then taken up by nearby pericytes or vascular smooth muscle cells promoting vasodilatation, an early step of angiogenesis (112). Extracellular nitric oxide also enters macrophages, increasing VEGF production by these cells (69). Consistent with their inhibitory effect on AKT, the HIV-protease inhibitors nelfinavir or ritonavir (and saquinavir, lopinavir, atazanavir, or amprenavir likewise) reduces eNOS expression and nitric oxide production in both endothelial and vascular smooth muscle cells (58, 79, 84, 119) (Figure 1, Table 2). As for HIV-protease inhibitors, the HIV-reverse transcriptase inhibitors zidovudine or lamivudine hinders eNOS, while stavudine has no effect (103) (Figure 1, Table 3). In contrast, emtricitabine, tenofovir or efavirenz stimulate eNOS (6) (Table 3).

Another downstream target of AKT important for angiogenesis is the mammalian-target-of-rapamycin (mTOR), a serine/threonine protein kinase which positively regulates HIF-1α protein (112) (Figure 1). Again in accord with their inhibitory effect on AKT, either nelfinavir or ritonavir can hamper mTOR activity in normal or cancer cells (23, 74, 85, 86, 91, 120–122) (Figure 1, Table 2).

## Impact of the HIV-Protease Inhibitors or HIV-Reverse Transcriptase Inhibitors on the Sequential Phases of Angiogenesis

**Figure 2:**
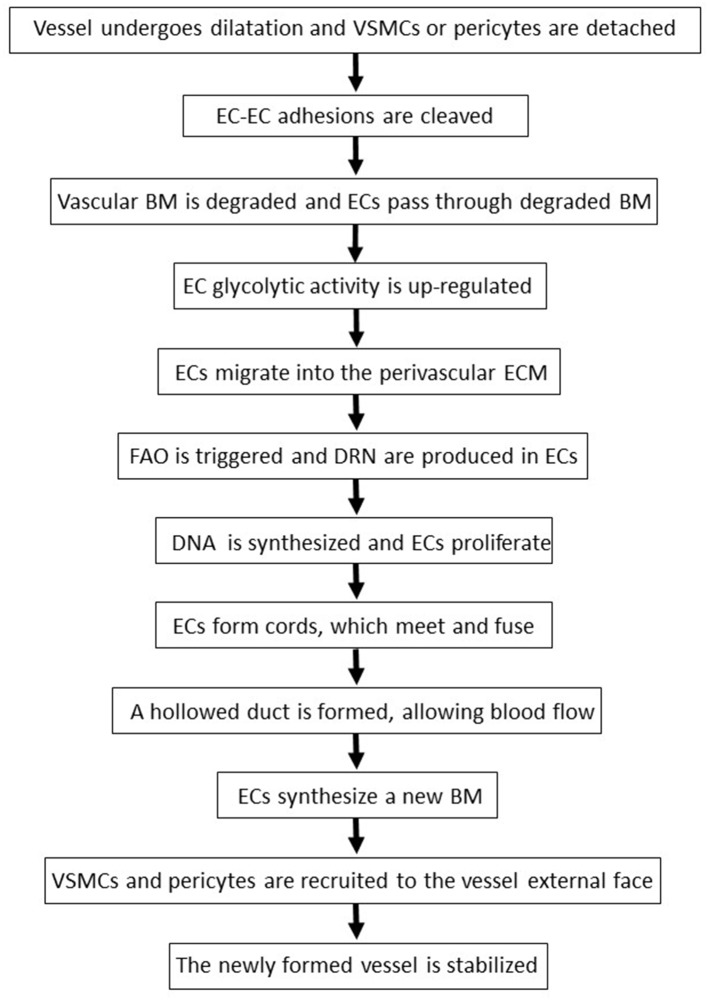
The sequential phases of angiogenesis. BM, basement membrane; DRN, deoxyribonucleotides; EC, endothelial cell; ECM, extracellular matrix; FAO, fatty acids oxidation; VSMC, vascular smooth muscle cells.

Angiogenesis is initiated by VEGF-A and angiopoietin-2 which, by activating AKT and MAPK, induce endothelial cells to secrete proteolytic enzymes such as the matrix metalloproteinases (MMPs): the latter detach vascular smooth muscle cells or pericytes from the vessels, cleave endothelial cell-endothelial cell adhesions and degrade the vascular basement membrane, releasing angiogenic molecules which are tethered therein (114, 115) (Figure 2).

Among MMPs, MMP-2 and MMP-9 levels are positively correlated to tumor vessel density (123). Either enzymes are synthesized as zymogens: pro-MMP-2 is activated on endothelial cell surface by membrane type 1-MMP, while pro-MMP-9 is turned-on by plasmin, outside the producing cells (123). Noteworthy, the HIV-protease inhibitor indinavir blocks MMP-2 activation in endothelial cells via a reduction of membrane type 1-MMP expression: this is due to indinavir capability of inhibiting the binding of the Specificity Protein-1 transcription factor to the promoter region of *membrane type 1-mmp* gene (57). About MMP-9, HIV-protease inhibitors including darunavir, lopinavir, saquinavir, and ritonavir directly down-regulate its expression in normal or tumor cells and in treated patients (28, 29, 80, 97, 100, 124) (Figure 1, Table 2). In particular, by inhibiting AKT phosphorylation, saquinavir or ritonavir impairs the activation of Fra-1, a transcription factor inducing MMP-9 expression (29).

In addition to be controlled at the transcriptional level and by zymogen activation, the function of MMPs is modulated by endogenous inhibitors, the so called Tissue Inhibitors of MMPs (TIMPs): in particular, the ratio between MMPs and TIMPs rules the intensity of vascular basement membrane or perivascular matrix degradation, thereby deeply influencing the angiogenic process (125). Noteworthy, the HIV-protease inhibitors nelfinavir, indinavir and ritonavir augment the levels of anti-angiogenic TIMPs either *in vitro* or in treated individuals (75, 124) (Figure 1, Table 2). This important finding is in agreement with the results from pre-clinical studies conducted with other antagonists of the MAPK/ERK or PI3K/AKT pathway (126, 127).

Definitely, the inhibitory effect on MMP activity provides a molecular explanation for indinavir, saquinavir, or amprenavir capability of impairing angiogenesis and tumor growth in HIV-free animal models (27, 52, 57, 99).

Concerning HIV-reverse transcriptase inhibitors, zidovudine down-regulates MMPs in astrocytes or microglia cells (104), but not in endothelial cells (105) (Figure 1); whereas, lamivudine increases MMP and decreases TIMP serum levels in treated individuals (108) (Table 3).

Following the degradation of the blood vessel basement membrane, endothelial cells migrate into the perivascular extracellular matrix in response to VEGF-A and other angiogenic factors (69, 123) (Figure 2). The locomotion of endothelial cells depends on their adhesion to extracellular matrix molecules such as collagen-I and fibronectin, which trigger endothelial cell survival and proliferation by binding to integrin receptors expressed on the surface of these cells (69). Interestingly, indinavir impairs cellular adhesion onto fibronectin (128), while nelfinavir or atazanavir reduce collagen deposition in the extracellular matrix (98, 129).

When exposed to high VEGF-A levels, endothelial cells remodel their actin cytoskeleton acquiring a migratory phenotype characterized by filopodia or lamellipodia (70). This differentiation process requires high levels of ATP which are produced via the up-regulation of endothelial cell glycolytic activity (70) (Figure 2). Specifically, by activating AKT, VEGF increases the expression of either glucose transporter-1, which promotes glucose entry into endothelial cells, or glycolytic enzymes, including lactate dehydrogenase A (70). Most of the glucose entering endothelial cells is then converted into lactate which, in turn, stabilizes HIF-1α, this increasing VEGF synthesis by endothelial cells and promoting their migration (70). Of interest, the HIV-protease inhibitor ritonavir potently reduces glucose transporter-1 activity and glycolysis (87) (Figure 1, Table 2). In contrast, the HIV-reverse transcriptase inhibitors efavirenz or zidovudine stimulates glycolysis and increases lactate levels (106, 110) (Figure 1, Table 3).

Upon VEGF binding to type 2 VEGF receptor, migratory endothelial cells secrete the Delta-like ligand 4 which binds to the Notch receptor on the surface of neighboring endothelial cells: the latter then proliferate, maintaining connectivity with migratory endothelial cells (69). In proliferating endothelial cells, the production of deoxyribonucleotides required for DNA synthesis is sustained by the oxidation of fatty acids (70) (Figure 2). Noteworthy, fatty acids oxidation is blocked by the HIV-protease inhibitors ritonavir, atazanavir, lopinavir or darunavir, and augmented by the HIV-reverse transcriptase inhibitors zidovudine and stavudine (88, 107) (Figure 1, Tables 2, 3).

Angiogenesis is terminated when newly formed vessels meet and fuse, giving rise to a hollowed duct which allows blood flow (69) (Figure 2). Thereafter, endothelial cells stabilize the vessels by synthesizing a new basement membrane and releasing platelet-derived growth factor-BB which, in turn, recruits mural vascular smooth muscle cells and pericytes (69) (Figure 2).

In this regard, it has to be highlighted that tumor vessels display a poor vascular smooth muscle cell coverage, and that tumor endothelial cells are more responsive to growth/chemotactic factors, and more resistant to apoptosis than normal endothelial cells (70, 114, 130). Tumors are consequently provided with overgrowing, highly permeable, dilated and tortuous vessels (70, 130). As this can compromise either the arrival of chemotherapeutics to the tumor site or the efficacy of anti-cancer radiotherapy (130–132), novel anti-angiogenesis strategies are aimed at remodeling the tumor vasculature to a more normal (and functional) phenotype. In this regard, atazanavir capability of down-regulating the oxygen-sensor-prolyl-hydroxylase-domain-protein 2 (98) suggests that this HIV-protease inhibitor could normalize tumor vessels. In fact, a decrease in prolyl-hydroxylase-domain-protein 2 switches tumor endothelial cell phenotype from migratory/proliferative to quiescent (130). In contrast, the HIV-protease inhibitors amprenavir, nelfinavir and ritonavir inhibit glycogen-synthase-kinase 3β (119, 133), an enzyme that favors the differentiation of tumor endothelial cells into normal endothelial cells (134). Moreover, ritonavir also hinders the growth and migration of vascular smooth muscle cells promoted by platelet-derived growth factor-BB (135).

Regarding HIV-reverse transcriptase inhibitors, efavirenz increases vessel permeability by altering endothelial cell-endothelial cell junctions (102).

In conclusion, results from the reviewed literature indicate that both HIV-protease inhibitors and HIV-reverse transcriptase inhibitors mainly hamper the initial steps of angiogenesis. However, one should consider that a block of endothelial cell invasion such that caused by HIV-protease inhibitors is enough to halt the whole angiogenic process (57, 114).

## Impact of HIV-Protease Inhibitors and Chemokine Receptor Antagonists on Vasculogenesis

The formation of tumor-associated vessels is facilitated and accelerated by vasculogenesis, a process which generally takes place during embryo life, involving the endothelial cell precursors: the latter are bone-marrow resident immature cells which, as for mature endothelial cells express both the type 2 VEGF receptor and the chemokine receptor CXCR4 (68, 136).

**Figure 3:**
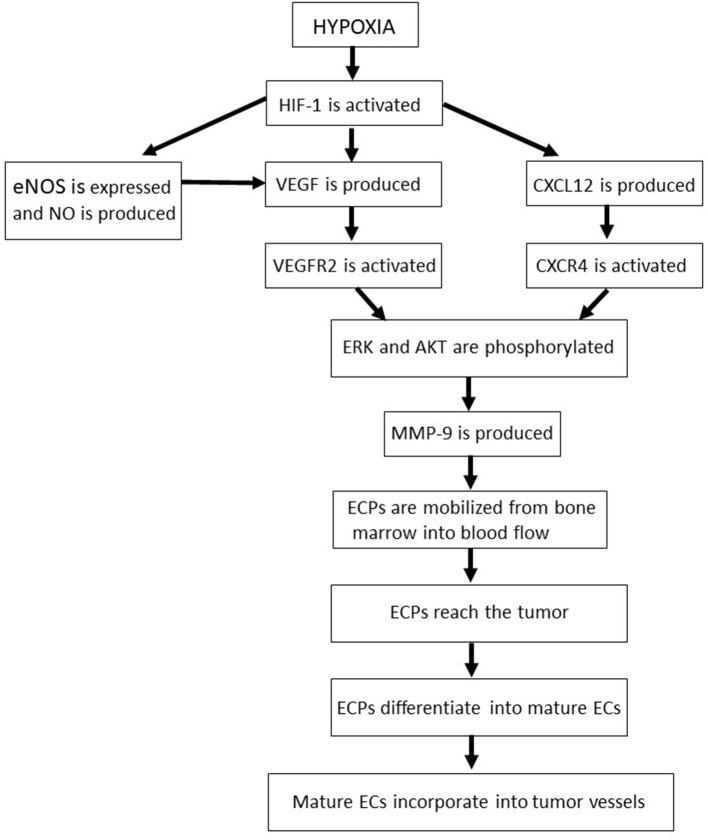
The molecular pathways and sequential phases of vasculogenesis. AKT, protein kinase B; CXCL12, CXC chemokine ligand 12; CXCR4, CXC chemokine receptor 4; EC, endothelial cell; ECP, endothelial cell precursor; eNOS, endothelial nitric oxide synthase; ERK, extracellular-signal-regulated kinases; HIF, hypoxia-inducible factor; MMP, matrix metalloproteinase; NO, nitric oxide; VEGF, vascular endothelial growth factor; VEGFR2, type 2 vascular endothelial growth factor receptor.

In tumor tissues, the activation of HIF-1 induces cancer cells, endothelial cells and fibroblasts to produce and release the angiogenic VEGF and CXCL12 (68, 69, 136) (Figure 3). In this context, nitric oxide bolsters VEGF production and release by endothelial cells (69) (Figure 3). Then, VEGF and CXCL12 reach the bone marrow and bind to type 2 VEGF receptor and CXCR4, respectively, on the surface of endothelial cell precursors (68, 69, 136) (Figure 3). Either the VEGF/type 2 VEGF receptor or the CXCL12/ CXCR4 axis triggers MAPK or AKT, sustaining the survival of endothelial cell precursors and activating MMP-9 production by those immature cells (68, 69, 136, 137) (Figure 3). MMP-9, in turn, mobilizes endothelial cell precursors from the bone marrow into the circulation, allowing them to reach the tumor (136) (Figure 3). There, because of the prolonged AKT activation triggered by VEGF, basic fibroblast growth factor and integrin-mediated adhesion onto tumor extracellular matrix, endothelial cell precursors differentiate into mature endothelial cells, then incorporating into tumor vessels (136) (Figure 3).

Consistent with CXCR4 role in the mobilization and survival of endothelial cell precursors, AMD3100, an antagonist of this receptor administered to HIV-infected individuals, effectively impairs tumor vasculogenesis in pre-clinical models (68, 111). Moreover, as activation of the CXCL12/CXCR4 axis stimulates VEGF and MMP production in mature endothelial cells (138), CXCR4 antagonists also inhibit tumor angiogenesis in animal models of human tumors (68).

Certainly, HIV-protease inhibitors possess all the features useful to counter tumor vasculogenesis, since they down-regulate HIF-1, AKT, MAPK, eNOS, VEGF and MMP (Figure 1, Table 2). Moreover, because of their inhibitory effect on glycogen-synthase-kinase 3β (119, 133), the HIV-protease inhibitors amprenavir, nelfinavir or ritonavir could hinder the differentiation of endothelial cell precursors into mature endothelial cells (139).

Concerning HIV-reverse transcriptase inhibitors, those stimulating eNOS activity, glycolysis and/or fatty acids oxidation (6, 106, 107, 110) (Figure 1, Table 3) could favor tumor-associated vasculogenesis. Future work will verify this possibility.

## Clinical Assessment of the Anti-Tumor Activity of Anti-HIV Drugs

Based on the results of pre-clinical and epidemiological studies, clinical trials have evaluated the effects of HIV-protease inhibitors, HIV-reverse transcriptase inhibitors or chemokine receptor antagonists (alone or combined with standard anti-tumor chemotherapy and/or radiotherapy) against a variety of tumors.

Results from medical tests performed in either HIV-positive or HIV-negative patients, indicate that HIV-protease inhibitor mono-therapy is effective against early-stage tumors including the angiogenic phase of Kaposi's sarcoma (10, 140, 141) and the pre-invasive stage of uterine cervical carcinoma (11) (Table 4). In contrast, when HIV-protease inhibitors are administered alone, they are not active against highly progressed tumors such as invasive uterine cervical carcinoma (8) and recurrent gliomas or adenoid cystic carcinomas (157, 158).

**Table 4:** Clinical outcomes and toxicities of anti-HIV drugs endowed with anti-tumor activities.

**Drug**	**Class**	**Positive outcomes**	**Toxicities**
Nelfinavir	HIV-PI	Colorectal or pancreas carcinoma, multiple myeloma (12–14)	Nausea, diarrhea, rash, changes in body fat distribution, hyperlipidemia, insulin resistance (4, 142, 143)
Indinavir	HIV-PI	Kaposi's sarcoma (7, 10)	Nausea, vomiting, diarrhea, abdominal pain, kidney stones (4, 142, 143)
Ritonavir	HIV-PI	Kaposis's sarcoma, uterine CIN, multiple myeloma (11, 15, 140)	Nausea, vomiting, diarrhea, abdominal pain, hyperlipidemia, insulin resistance (4, 142, 143)
Lopinavir	HIV-PI	Uterine CIN (11)	Nausea, vomiting, diarrhea, tendinopathy, insulin resistance (4, 142, 143)
Efavirenz	HIV-RTI	Prostate carcinoma (16)	Headache, nausea, rash, neuropsychiatric disorders, seizures (3, 144)
Zidovudine	HIV-RTI	Kaposi's sarcoma, T cell leukemia/lymphoma, EBV-related lymphoma, Castleman disease (17–20, 145, 146)	Headache, nausea, vomiting, neutropenia, anemia, hepatotoxicity, myopathy (147)
Tenofovir	HIV-RTI	Hepatocellular carcinoma (21, 148)	Headache, nausea, diarrhea, rash, nephrotoxicity (3, 149)
Lamivudine	HIV-RTI	Hepatocellular carcinoma (21, 150, 151)	Headache, nausea, vomiting, diarrhea, neutropenia, anemia, myopathy (3, 152)
AMD3100	CXCR4 antagonist	Acute myeloid leukemia (9, 153–155)	Headache, nausea, vomiting, diarrhea (156)

*Here are summarized the positive clinical oncological outcomes and the toxicities of the inhibitors of HIV protease or reverse transcriptase, and the AMD3100 antagonist of CXCR4, employed in either HIV-positive or HIV-negative patients. CIN, cervical intraepithelial neoplasia; EBV, Epstein-Barr virus; HIV-PI, HIV-protease inhibitor; HIV-RTI, HIV-reverse transcriptase inhibitor; NSCLC, non-small cells lung carcinoma. In square brackets are references with specific information*.

Concerning the combination of HIV-protease inhibitors and classical anti-cancer radiotherapy and/or chemotherapy, the great majority of clinical trials has been performed for nelfinavir. Results from these trials indicate that, in addition to be safe and well-tolerated, combination therapy with nelfinavir is associated with the regression of rectal or pancreatic carcinoma (Table 4), this being accompanied by the “normalization” of the aberrant tumor vasculature (12, 13). In addition, nelfinavir has also been reported to overcome the resistance of multiple myeloma to proteasome inhibitors (14) (Table 4).

Regarding ritonavir-based or saquinavir-based combinatory therapies, the inhibitory effect that these HIV-protease inhibitors exert on cytochrome P450 has been shown to impair the clearance of anti-cancer chemotherapeutics: this has led to severe toxicities in some clinical trials (159–161), but not in others (15).

Concerning the toxicity that HIV-protease inhibitors have in themselves, results from large observational studies conducted on HIV-infected subjects, indicate that HIV-protease inhibitor-based cART elevates cholesterol, triglycerides and glucose plasma levels (142, 143). These side effects have occurred mostly with first generation HIV-protease inhibitors such as ritonavir, nelfinavir or lopinavir (Table 4), which increase lipemia by inhibiting adiponectin expression, and/or cause insulin resistance via an impairment of glucose transporter-4 activity (4). In contrast, second generation HIV-protease inhibitors including darunavir and atazanavir display only minor toxicities and have no effect on lipemia or insulin sensitivity (4).

Regarding the HIV-reverse transcriptase inhibitors, most of the clinical trials have been performed for zidovudine. Results indicate that when combined with antivirals (e.g., interferon α or valgaciclovir) or cytotoxic drugs (e.g., bleomycin or methotrexate), zidovudine is effective against HIV-associated Kaposi's sarcoma (145), T cell leukemia (17, 18, 20), Epstein-Barr virus related-lymphomas (19) or Castleman lymphoproliferative disease (146) (Table 4). Other clinical studies have shown that hepatitis B virus-infected cancer patients have a reduced incidence of hepatocellular carcinoma when they undergo prophylactic treatment with HIV reverse transcriptase inhibitors including lamivudine, entecavir or tenofovir (21, 148, 150, 151) (Table 4). Finally, high plasma concentrations of efavirenz have been reported to slow the progression of prostate carcinoma (16) (Table 4).

However, in the majority of the abovementioned diseases, the anti-tumor activity of HIV-reverse transcriptase inhibitors is not likely to depend on the impairment of angiogenesis, but rather on the capability that these anti-HIV drugs have to hamper the replication of tumorigenic viruses (17–21, 145, 146, 148, 150, 151).

Observational studies have shown that the most common adverse effects which can occur in HIV-infected patients taking HIV-reverse transcriptase inhibitors include headache, nausea, vomiting, diarrhea and/or rash (147, 149, 152) (Table 4). In addition, long-term use or high-dose treatment with HIV-reverse transcriptase inhibitors is associated with severe, therapy-limiting side effects such as anemia, neutropenia, myopathy, impaired liver function or nephrotoxicity (147, 149, 152) (Table 4). Furthermore, some of the efavirenz-treated patients have experienced neuropsychiatric disorders and/or seizures (144) (Table 4).

About the use of chemokine receptor antagonists in cancer patients, the CXCR4 antagonist AMD3100 is the only one approved for clinical use: at the present time, the drug is being employed mostly in order to mobilize hematopoietic stem cells from the bone marrow, hence allowing their autologous transplantation in patients affected by non-Hodgkin lymphoma or multiple myeloma (153, 154). In addition, AMD3100 has recently been used to mobilize bone marrow-resident leukemic cells into the blood flow, this leading to the sensitization of leukemic cells to cytotoxic drugs (155) (Table 4). Interestingly, results from pre-clinical studies support AMD3100 combination with radio/chemotherapy to counteract lymph node or distant metastases of solid tumors (38, 48, 162, 163). This strategy is also sustained by observational studies (164, 165).

Headache, nausea, vomiting, diarrhea or muscle/bone pains are relatively frequent in patients treated with AMD3100 (Table 4): however, in most cases these adverse effects are mild and transient (156).

## Classical Anti-Angiogenic Drugs: The VEGF Antagonists

Combining angiogenesis antagonists with standard anti-tumor therapies has ameliorated the prognosis of a variety of human tumors (70).

In particular, inhibitors of the VEGF pathway have provided survival benefits to cancer patients (114).

At the present time, the VEGF antagonists which have been approved for clinical use include: (a) BEVACIZUMAB, a recombinant humanized monoclonal antibody which binds to VEGF-A and prevents its interaction with the type 2 VEGF receptor (166); (b) RAMUCIRUMAB, a blocking antibody directed against type-2 VEGF receptor that impedes its binding by VEGF (167); (c) AFLIBERCEPT, a human recombinant fusion protein that acts as a soluble decoy receptor sequestering various members of the VEGF family (168); (d) the inhibitors of receptor kinase activity, small molecules which pass through the cell membrane and prevent ATP binding to type-2 VEGF receptor (169) (Table 5).

**Table 5:** Indications and toxicities of anti-VEGF compounds approved for clinical use.

**Drug**	**Mechanism of action**	**Indication(s)**	**Toxicities**
BEVACIZUMAB	Humanized antibody which binds to VEGF-A, preventing its binding to VEGF receptor (166)	Mesothelioma, NSCLC, or colorectal, ovarian, cervical, renal carcinoma (166, 170–174)	Hypertension, neutropenia, proteinuria, rash, bleeding, thromboembolism, fistula (69, 175–178)
AFLIBERCEPT	Recombinant fusion protein that sequesters VEGF (168)	Colorectal carcinoma (168)	Hypertension, neutropenia, lymphopenia, thrombocytopenia, thromboembolism (69, 179)
RAMUCIRUMAB	Anti-VEGFR2 antibody which competes VEGF binding (167)	NSCLC, or colorectal, esophageal, gastric carcinoma (167, 180, 181)	Hypertension, neutropenia, anemia, thrombocytopenia, proteinuria, bleeding, gastrointestinal perforation, wound healing complications (69, 175, 176, 178, 182)
SUNITINIB	Small peptide that prevents ATP binding to VEGFR2 (169)	NSCLC, or colorectal, pancreas, renal carcinoma (169)	Hypertension, neutropenia, thrombocytopenia, rash, gastrointestinal perforation (69, 176, 178, 183, 184)

*Here are summarized the mechanism of action, the oncological indications and the toxicities of the inhibitors of the vascular endothelial growth factor pathway approved for clinical use. ATP, adenosine triphosphate; NSCLC, non-small cells lung carcinoma; VEGF, vascular endothelial growth factor; VEGFR2, type 2 vascular endothelial growth factor receptor. In square brackets are references with specific information*.

Results from clinical trials combining VEGF pathway inhibitors and standard anti-tumor cytotoxic drugs indicate that BEVACIZUMAB augments overall survival and/or progression-free survival in patients affected by mesothelioma, non-small cell lung cancer or colon, renal, ovarian or uterine cervical carcinoma (166, 170–174), while RAMUCIRUMAB provides survival benefits in individuals with gastric cancer, non-small cell lung cancer or colon carcinoma (167, 180, 181) (Table 5). For their part, either AFLIBERCEPT or the receptor kinase inhibitors SUNITINIB and PAZOPANIB are effective against colorectal carcinoma (168, 169) (Table 5).

However, long-term use of VEGF antagonists can lead to side effects including bone marrow toxicity (this resulting in neutropenia and, eventually, lymphopenia, thrombocythemia and/or anemia), hypertension, proteinuria, liver malfunction, diarrhea, gastrointestinal perforations, reduced wound healing, disturbances in blood clotting, skin rash or discoloration, hyponatremia and/or hyperglycemia (69, 175–179, 182–184) (Table 5). Moreover, VEGF inhibitors trigger hypoxia, which exacerbates tumor aggressiveness (70). In addition, treated patients can develop drug resistance over time: this occurs via the up-regulation of the angiogenic basic fibroblast growth factor, angiopoietin-2 or CXCL12 (68).

Altogether, these clinical findings are stimulating the continual search for other antagonists of angiogenesis.

## Conclusions and Perspectives

Cancer growth and dissemination are favored and sustained by the formation of new blood vessels within the tumor area (69, 70, 114). Consistently, compounds counteracting the development of tumor vasculature can limit or slow cancer clinical progression (69, 70, 114).

In this regard, antagonists of the highly angiogenic VEGF have been found effective against different types of human tumors (166–174, 180, 181) (Table 5).

However, the finding that patients treated with these drugs can undergo severe adverse effects (69, 70, 175–179, 182–184) (Table 5), and/or develop drug resistance (68), is prompting the identification of anti-angiogenesis drugs alternative to VEGF antagonists.

In this context, it has to reminded that clinical trials assessing the anti-tumor efficacy of synthetic MMP inhibitors impairing tumor angiogenesis in pre-clinical models have failed: this has been due to the poor solubility, lack of specificity and/or inefficacy of the drugs (123).

In view of chemokine role in inflammation-driven, pathological angiogenesis, antagonists of the chemokine receptors are currently being evaluated for their efficacy in countering tumor growth and metastases (69). Indeed, drugs targeting single chemokine receptors have been found effective against hematological malignancies (9, 153, 154). Moreover, CXCR4 antagonists have been shown to reduce tumor angiogenesis in animal models of human tumors (68). However, one should consider that a given chemokine can bind to different receptors, which are all capable of triggering the AKT-MMP pathway starting angiogenesis (136). In this regard, HIV-protease inhibitors effectively inhibit the AKT-MMP as well as other pathways which lead to both new blood vessel formation (Figure 1, Table 2) and cancer cell survival, growth or locomotion (7, 8).

In contrast, based on the reviewed literature, the anti-angiogenic actions of HIV-reverse transcriptase inhibitors appear limited: in fact, these drugs hamper some pro-angiogenic pathways, while favoring others (Figure 1, Table 3).

Among HIV-protease inhibitors, ritonavir and nelfinavir have proven to be particularly effective in inhibiting tumor-associated new blood vessel formation (Figure 1, Table 2). Repositioning of these anti-HIV drugs in cancer therapy has been feasible, as they are employed since many years, and their pharmacokinetic and tissue distribution are well known (4). Actually, clinical trials combining ritonavir or nelfinavir with standard anti-cancer therapeutics have given good results (13–15). However, as for other first-generation HIV-protease inhibitors, either nelfinavir or ritonavir increases lipid and glucose plasma levels in treated patients (4, 142, 143). Though novel HIV-protease inhibitors such as darunavir and atazanavir do not affect lipemia or glycemia (4), information on their anti-angiogenic activities is narrow (Table 2).

Therefore, added work should dissect darunavir or atazanavir impact on angiogenesis, and then design and test atazanavir, darunavir, ritonavir or nelfinavir analogs endowed with selective anti-angiogenic effects. To this end, further molecular modeling approaches and protein-ligand studies are needed in order to identify more precisely the targets of HIV-protease inhibitors.

Finally, given that inflammation plays a major role in new blood vessel formation (69, 70), additional clinical investigations should evaluate whether the anti-angiogenic effect of anti-HIV drugs could be potentiated by anti-inflammatory drugs, including cyclooxygenase 2 inhibitors which have been shown to promote tumor vessel normalization in pre-clinical models (185).

## Author Contributions

The author confirms being the sole contributor of this work and has approved it for publication.

## Conflict of Interest

The author declares that the research was conducted in the absence of any commercial or financial relationships that could be construed as a potential conflict of interest.

## Abbreviations

AIDS: Acquired Immune Deficiency Syndrome
cART: combined Anti-Retroviral Therapy
CXCL12: CXC chemokine ligand 12
CXCR4: CXC chemokine receptor 4
eNOS: endothelial nitric oxide synthase
HIF: hypoxia inducible transcription factor
HIV: human immunodeficiency virus
MAPK/ERK: mitogen-activated protein kinases/extracellular-signal-regulated kinases
MMP: matrix metalloproteinase
NF-kB: Nuclear Factor-kappa B
PI3K/AKT: phosphoinositide3-kinase/protein kinases B
TIMP: tissue inhibitor of matrix metalloproteinase
VEGF: vascular endothelial growth factor.
